# 1693. Incidence of Pneumococcal Disease in Children ≤48 Months Old in the United States: 2010-2019

**DOI:** 10.1093/ofid/ofad500.1526

**Published:** 2023-11-27

**Authors:** Salini Mohanty, Nicolae Done, Qing liu, Yan Song, Katherine Gaburo, Travis Wang, Eric M Sarpong, Meghan White, Jessica P Weaver, James Signorovitch, Thomas Weiss

**Affiliations:** Merck & Co., Inc, Rahway, New Jersey; Analysis Group, Boston, Massachusetts; Analysis Group, Boston, Massachusetts; Analysis Group, Inc., Boston, Massachusetts, USA, Boston, MA; Analysis Group, Inc., Boston, Massachusetts; Analysis Group, Inc., Boston, Massachusetts; Merck & Co., Inc., Rahway, New Jersey; Merck & Co., Inc., Rahway, New Jersey; Merck & Co., Inc., Rahway, New Jersey; Analysis Group, Boston, Massachusetts; Merck & Co., Inc., Rahway, New Jersey

## Abstract

**Background:**

Pneumococcal disease (PD) leads to considerable morbidity, mortality, and healthcare resource utilization in children. This study estimated incidence rates (IRs) for invasive PD (IPD), all-cause pneumonia (ACP), and acute otitis media (AOM) among children ≤ 48 months old, between 2010 and 2019 in the US.

**Methods:**

This was a retrospective observational study using MarketScan^®^ Commercial Claims and Encounters (CCAE) and Multi-State Medicaid databases (2010-2019). Date of birth was imputed using dates of live-birth claims for accurate age determination. IPD, ACP, and AOM claims in children ≤48 months old were identified using International Classification of Diseases (ICD) versions 9 and 10 diagnosis codes. Episodes were defined as one or more PD-related claims, with a gap of 90 days between two IPD or ACP claims, and 14 days between two AOM claims. IRs were defined as number of episodes per 100,000 person-years (PY) for IPD and ACP, and per 1,000 PY for AOM. Annual IRs were stratified by age (0-6, 7-12, 13-24, and 25-48 months), and reported separately for CCAE and Medicaid populations.

**Results:**

Overall IPD IRs declined between 2010-2019 from 11 to 7 episodes per 100,000 PY in CCAE and from 20 to 9/100,000 PY in Medicaid; IPD IRs were highest in children 0-6 months old, followed by 7-12 months old, in both populations (**Fig. 1**). Overall ACP IRs changed little between 2010-2019 from 3,996 to 3,952/100,000 PY in CCAE and declined from 6,225 to 4,521 per 100,000 PY in Medicaid; ACP IRs were highest in CCAE among children aged 13-24 and 25-48 months, and in Medicaid among children aged 13-24 and 7-12 months. (**Fig. 2**). Overall AOM IRs changed little between 2010-2019 from 723 to 738/1,000 PY in 2018 in CCAE and decreased from 773 to 624/1,000 PY in Medicaid (**Fig. 3**). AOM IRs were highest for children 7-12 months in both populations.

**Figure 1.**
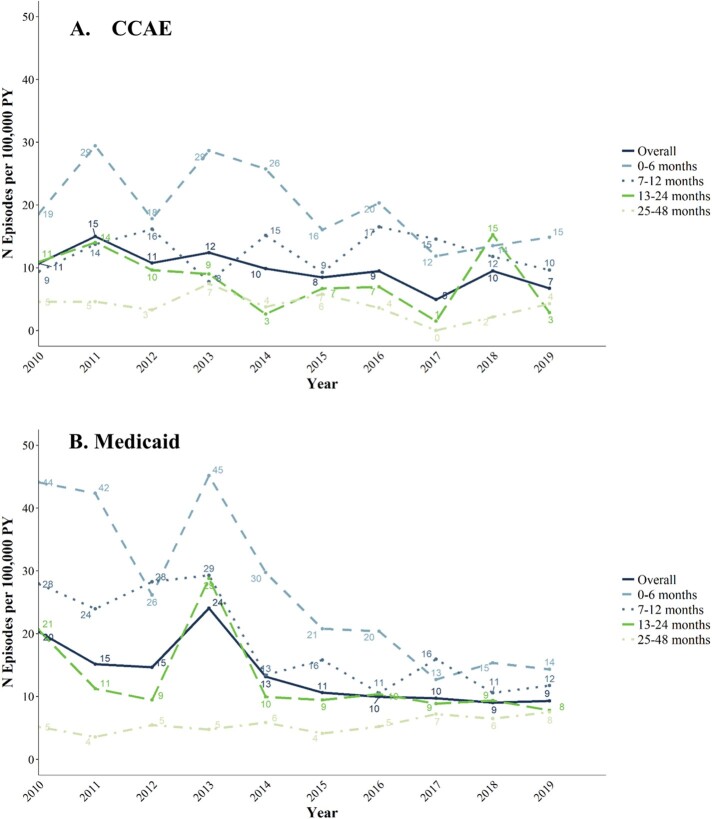
IPD incidence in commercially and Medicaid-insured children ages 0 - 48 months, episodes per 100,000 patient-years (2010 - 2019)

**Figure 2.**
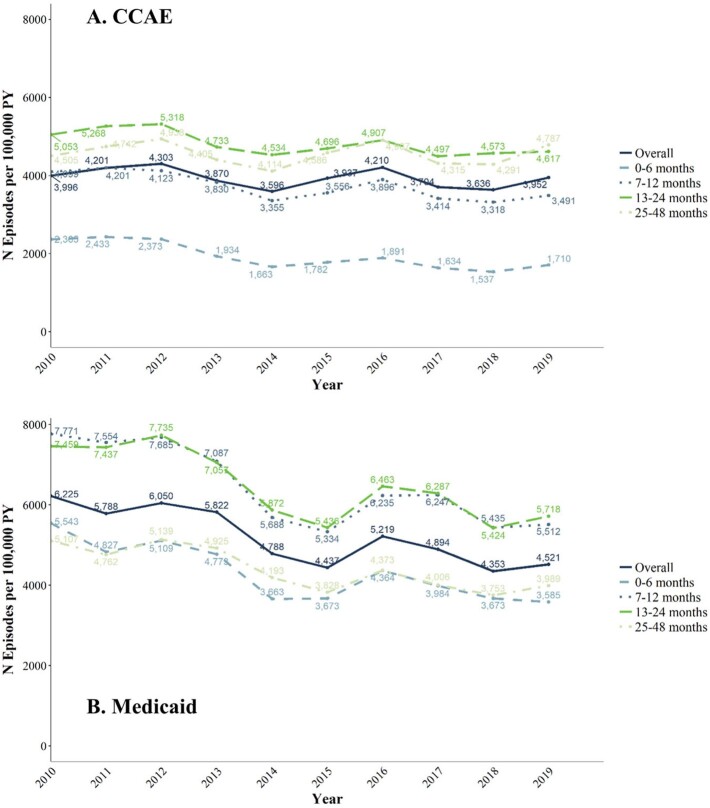
ACP incidence in commercially and Medicaid-insured children ages 0 - 48 months, episodes per 100,000 patient-years (2010 - 2019)

**Figure 3.**
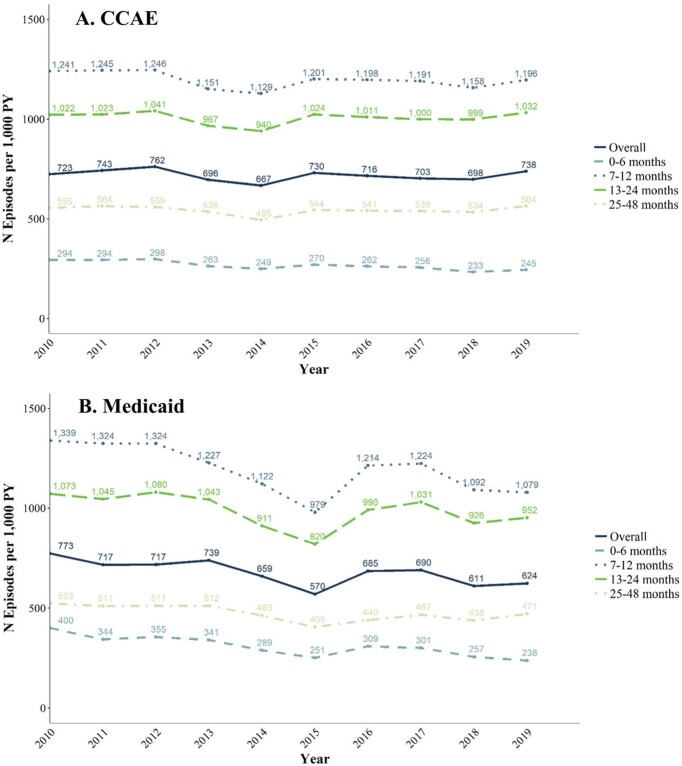
AOM incidence in commercially and Medicaid-insured children ages 0 - 48 months, episodes per 1,000 patient-years (2010 - 2019)

**Conclusion:**

IPD, ACP, and AOM IRs disease burden remains substantial with a disproportionately high impact of the most severe disease manifestations (IPD and inpatient ACP) among infants in the first year of life, despite marked declines following the introduction of PCV13. Disease IRs were generally higher in Medicaid children, particularly at the start of the study period, but also declined more significantly over time compared to IRs in CCAE children.

**Disclosures:**

**Salini Mohanty, DrPH, MPH**, Employee of Merck & Co., Inc.: Stocks/Bonds **Nicolae Done, PhD**, Merck & Co., Inc.: Grant/Research Support **Qing liu, PhD**, Merck & Co., Inc.: Advisor/Consultant **Yan Song, PhD**, Merck & Co., Inc.: Grant/Research Support **Katherine Gaburo, n/a**, Merck & Co., Inc.: I am an employee of Analysis Group, Inc., which received consulting fees for participation in this research **Travis Wang, MS, MBBS**, Merck & Co., Inc.: Grant/Research Support **Meghan White, PharmD**, Merck: Employee|Merck: Stocks/Bonds **Jessica P. Weaver, PhD**, Employee of Merck & Co., Inc.: Stocks/Bonds **James Signorovitch, PhD**, Merck & Co., Inc.: Grant/Research Support **Thomas Weiss, DrPH, MPH**, Employee of Merck & Co., Inc.: Stocks/Bonds

